# Enhancement of Gamma-ray Shielding Properties in Cobalt-Doped Heavy Metal Borate Glasses: The Role of Lanthanum Oxide Reinforcement

**DOI:** 10.3390/ma14247703

**Published:** 2021-12-13

**Authors:** Ghada ALMisned, Wiam Elshami, Shams A. M. Issa, Gulfem Susoy, Hesham M. H. Zakaly, Merfat Algethami, Y. S. Rammah, Antoaneta Ene, S. A. Al-Ghamdi, Awad A. Ibraheem, H. O. Tekin

**Affiliations:** 1 Department of Physics, College of Science, Princess Nourah Bint Abdulrahman University, Riyadh 11671, Saudi Arabia; gaalmisned@pnu.edu.sa; 2 Medical Diagnostic Imaging Department, College of Health Sciences, University of Sharjah, Sharjah 27272, United Arab Emirates; welshami@sharjah.ac.ae; 3 Physics Department, Faculty of Science, University of Tabuk, Tabuk 47512, Saudi Arabia; sh_issa@ut.edu.sa (S.A.M.I.); saalghamdi@ut.edu.sa (S.A.A.-G.); 4 Physics Department, Faculty of Science, Al-Azhar University, Assiut 71524, Egypt; 5 Department of Physics, Faculty of Science, Istanbul University, Istanbul 34134, Turkey; glfmsusoy972@gmail.com; 6 Institute of Physics and Technology, Ural Federal University, 620002 Ekaterinburg, Russia; 7 Physics Department, Faculty of Science, Taif University, P.O. Box 11099, Taif 21944, Saudi Arabia; M.algethami@tu.edu.sa; 8 Department of Physics, Faculty of Science, Menoufia University, Shebin El-Koom 32511, Egypt; dr_yasser1974@yahoo.com; 9 INPOLDE Research Center, Department of Chemistry, Physics and Environment, Faculty of Sciences and Environment, Dunarea de Jos University of Galati, 47 Domneasca Street, 800008 Galati, Romania; 10 Physics Department, King Khalid University, Abha 62529, Saudi Arabia; awad@gmail.com; 11 Medical Radiation Research Center (USMERA), Uskudar University, Istanbul 34672, Turkey

**Keywords:** lead borate glasses, gamma ray, mass attenuation, radiation resistance

## Abstract

The direct influence of La^3+^ ions on the gamma-ray shielding properties of cobalt-doped heavy metal borate glasses with the chemical formula 0.3CoO-(80-x)B_2_O_3_-19.7PbO-xLa_2_O_3_: x = 0, 0.5, 1, 1.5, and 2 mol% was examined herein. Several significant radiation shielding parameters were evaluated. The glass density was increased from 3.11 to 3.36 g/cm^3^ with increasing La^3+^ ion content from 0 to 2 mol%. The S5 glass sample, which contained the highest concentration of La^3+^ ions (2 mol%), had the maximum linear (μ) and mass (μ_m_) attenuation coefficients for all photon energies entering, while the S1 glass sample free of La^3+^ ions possessed the minimum values of μ and μ_m_. Both the half value layer (T_1/2_) and tenth value layer (TVL) of all investigated glasses showed a similar trend of (T_1/2_, TVL)_S1_ > (T_1/2_, TVL)_S2_ > (T_1/2_, TVL)_S3_ > (T_1/2_, TVL)_S4_ > (T_1/2_, TVL)_S5_. Our results revealed that the S5 sample had the highest effective atomic number (Z_eff_) values over the whole range of gamma-ray energy. S5 had the lowest exposure (EBF) and energy absorption (EABF) build-up factor values across the whole photon energy and penetration depth range. Our findings give a strong indication of the S5 sample’s superior gamma-ray shielding characteristics due to the highest contribution of lanthanum oxide.

## 1. Introduction

Gamma radiation is commonly used in medicine and in industry [1]. The biological effect of radiation is well known; consequently, massive efforts are being made in the field of radiation shielding. Lead-based shields were commonly used, but there is growing interest in other materials to reduce toxicity and improve shielding efficiency. Borate glass is a promising material in the field of radiation shielding and protection due to its chemical and physical properties. Oxides can be added to improve the optical features, mechanical characteristics, and shielding properties of borate glasses. Many studies have investigated the effect of adding Na_2_O, CaO, Nd_2_O_3_, and Gd_2_O_3_ to borate glass [2,3,4]. Similarly, the addition of lanthanum oxide (La_2_O_3_) showed an improvement in optical glass properties [5,6]. La_2_O_3_, commonly known as lanthana, is an odorless white powder that is slightly soluble in acids and water. La_2_O_3_ is an essential rare-earth compound used in many areas, such as optical glasses, fluorescent lamps, dielectric and conductive ceramics, and X-ray intensifying screens. Furthermore, La_2_O_3_ provides good process compatibility as it is not crystallized after heat treatment at temperatures up to 900 °C [7]. Because of its high dielectric constant (k ≈ 27) and relatively large band gap (E_g_ = 5.8 eV), La_2_O_3_ is well recognized as a gate dielectric material. On the other hand, La_2_O_3_ is hygroscopic, has poor thermal stability, and has poor interface properties. These factors reduce the k value and generate a positive fixed charge, resulting in deterioration of its dielectric properties [8,9]. Therefore, adding other elements to La_2_O_3_ will improve its properties; previous studies used silicon, aluminum, and nitrogen to enhance the chemical and electrical characteristics [10,11]. Nevertheless, La_2_O_3_ is a possible candidate for modifying the physical and optical properties of glasses and glass-ceramics. For example, La_2_O_3_ improves the water resistance of borate optical glasses. Lithium borate glasses have received the most attention because lithium has a promising future in high-energy-density batteries and other electrochemical applications due to its light weight and most electropositive nature [5,12].

Current studies in the literature clearly reveal that the scientific community is very interested in the manufacture of different types of oxide glasses. Characterization of these produced glasses in terms of critical material properties, such as radiation resistance, is a well-established technique for acquiring a better understanding of the nature of glass and its components, from the base to the reinforcing elements. In this research, several kinds of lanthanum-oxide-reinforced glasses with varying compositions were investigated:

S1: 0.3CoO-80B_2_O_3_-19.7PbO (ρ = 3.11 g/cm^3^);

S2: 0.3CoO-79.5B_2_O_3_-0.5La_2_O_3_-19.7PbO (ρ = 3.18 g/cm^3^);

S3: 0.3CoO-79B_2_O_3_-1La_2_O_3_-19.7PbO (ρ = 3.24 g/cm^3^);

S4: 0.3CoO-78.5B_2_O_3_-1.5La_2_O_3_-19.7PbO (ρ = 3.29 g/cm^3^);

S5: 0.3CoO-78B_2_O_3_-2La_2_O_3_-19.7PbO (ρ = 3.36 g/cm^3^).

The purpose of this paper was to examine the impact of an increasing quantity of lanthanum oxide reinforcement in the glass composition on the glass’s gamma-ray shielding capabilities. The conclusions of this investigation may be beneficial for the literature regarding glass, particularly for glassy alloys, including lanthanum oxide.

## 2. Materials and Methods

In this study, a group of lanthanum-oxide-reinforced heavy metal borate glasses with the chemical formula 0.3CoO-(80-x)B_2_O_3_-19.7PbO-xLa_2_O_3_: x = 0, 0.5, 1, 1.5, and 2 mol% were investigated in terms of their extensive gamma-ray shielding parameters. Previously, Abul-Magd et al. [13] studied the effect of the rare-earth compound lanthanum oxide on the structural, mechanical, and optical properties of a glassy system composed of cobalt-doped heavy metals. Our objective was to conduct a theoretical follow-up analysis on those glass samples in order to provide new skills and information to the scientific community by analyzing their gamma-ray shielding properties.

### 2.1. Fundamental Shielding Parameters

The linear attenuation coefficient term (μ) for photons of given energy in a certain material is the result of numerous physical processes that result in photon emission from the beam. A material’s linear attenuation coefficient is determined by its density. By normalizing the linear attenuation coefficient (μ) with the absorber density (ρ), the dependency on the absorber density is removed. Because it is independent of the absorber density and physical state, the mass attenuation coefficient defined by μ/ρ is more fundamental than the linear coefficient value. The photons are transmitted in accordance with the Beer–Lambert law when a gamma ray interacts with a sample of thickness x (cm) with respect to the narrow beam geometry [14,15].

(1) $$I=I_{0}e^{-\mu x}$$

The $I_{0}$ and I values in Equation (1) denote the gamma-ray intensity before and after passing through a sample of thickness x (cm). Further, μ (cm^−1^) is the linear attenuation coefficient of the sample and t (cm) is the physical thickness of the shielding material.

The linear attenuation coefficient can be defined from the point of mass attenuation coefficient as follows:

(2) $$\mu =\left(\;\frac{\mu \;}{\rho \;}\right)\rho ={\left(\mu \right)}_{s}\rho \;$$

where ${\left(\mu \right)}_{s}=\left(\;\frac{\mu \;}{\rho \;}\right)$ (cm^2^g^−1^) is the mass attenuation coefficient and ρ (g/cm^3^) is the density of the sample.

The mass attenuation coefficient of a composite or mixture is equal to the total of the weights of each of the constituent elements [16,17].

(3) $${\left(\mu \right)}_{s}={\left(\frac{\mu }{\rho }\right)}_{\mathrm{mix}}=\underset{i}{\sum }w_{i}{\left(\frac{\mu }{\rho }\right)}_{i}$$

This expression is the mixing rule, also known as Bragg’s rule. In this equation, $w_{i}$ is the weight fraction and ${\left(\mu /\rho \right)}_{i}$ is the mass attenuation coefficient of the *i*th element. The fraction by weight $w_{i}$ of a chemical compound is calculated using the equation below [18,19].

(4) $${\;w}_{i}=\frac{n_{i}A_{i}}{\sum_{i}n_{i}A_{i}}$$

The half value layer (HVL), tenth value layer (TVL), and mean free path (MFP) are the other essential metrics for determining a material’s shielding capabilities.

(5) $$\mathrm{HVL}=\frac{0.693}{\mu }$$

(6) $$\mathrm{TVL}=\frac{\ln10}{\mu }$$

(7) $$\mathrm{MFP}=\frac{1}{\mu }$$

The HVL and TVL refer to the material thicknesses that attenuate one-half and one-tenth of the photon strength, respectively. The MFP is the average distance that a photon with a certain energy can travel without any interaction [20,21].

Using the XCOM program, the $\left(\;\frac{\mu \;}{\rho \;}\right)$ values of the samples were calculated.

The direct technique was used to determine Z_eff_, which is a crucial parameter to be aware of when considering gamma protection properties [22,23]. The Z_eff_ value is the parameter measured to show the meaning of the gamma ray and x-ray material absorption fractions of materials prepared for radiation shielding, and it can be calculated from the following equation [24,25].

(8) $$Z_{\mathrm{eff}}=\frac{\sum_{i}f_{i}A_{i}{\left(\frac{\mu }{\rho }\right)}_{i}}{\sum_{j}\frac{f_{j}A_{j}}{Z_{\mathrm{ij}}}{\left(\frac{\mu }{\rho }\right)}_{j}}$$

In this equation, for the ith and jth elements, $f_{i}$ and $f_{j}\;$ are the respective fractional abundances according to the number of atoms, $Z_{i}$ and $Z_{j}$ denote the respective atomic numbers, and $A_{i}$ and $A_{j}$ are the respective weights [26,27].

Additionally, the effective electron density (N_eff_) can be estimated using Z_eff_ as follows:

(9) $$N_{\mathrm{eff}}=\frac{N_{A}}{M}Z_{\mathrm{eff}}\sum^{\;}n_{i}\;$$

where N_A_ is Avogadro’s number and M is the atomic mass of the glass.

In all areas where radiation is used or maintained, reliable and highly sensitive information about the EABF and EBF parameters is needed. The EBF value represents the degree of probable air interactions between the radiation source and the detector. EABF, on the other hand, is a parameter that refers to how much energy is absorbed in the substance with which the radiation interacts [28,29,30,31]. The EABF and EBF values of the examined glasses were obtained using the GP fitting approach, as previously reported in several papers [32,33,34]. These two parameters are almost equal in terms of determining the ratio of un-collided/un-scattered photons. The GP fitting parameters obtained by interpolation of the equivalent atomic number (Z_eq_) were used to calculate the EABF and EBF factors with the help of the following equations:

(10) $$Z_{\mathrm{eq}}=\frac{Z_{1}\left(\log R_{2}-\log R\right)+Z_{2}\left(\log R-\log R_{1}\right)}{\log R_{2}-\log{\;R}_{1}}$$

where Z_1_ and Z_2_ indicate the atomic numbers of the samples for the ratios R_1_ and R_2_, respectively, and

(11) $$C=\frac{C_{1}\left(\log Z_{2}-\log Z_{\mathrm{eq}}\right)+C_{2}\left(\log Z_{\mathrm{eq}}-\log Z_{1}\right)}{\log Z_{2}-\log Z_{1}}$$

where C_1_ and C_2_ denote the GP fitting parameters for the elements with atomic numbers Z_1_ and Z_2_, respectively.

### 2.2. Monte Carlo Simulations and Theoretical Calculations of Gamma-ray Shielding Properties

The mass attenuation coefficients of the S1, S2, S3, S4, and S5 glasses were effectively computed using the general-purpose Monte Carlo tool MCNPX (Los Alamos National Laboratory) [35] (version 2.7.0). First, input data for MCNPX were prepared using the following fundamental components:

- *Card for a cell*;
- *Card for a surface*;
- *Source data*.

**Figure 1:**
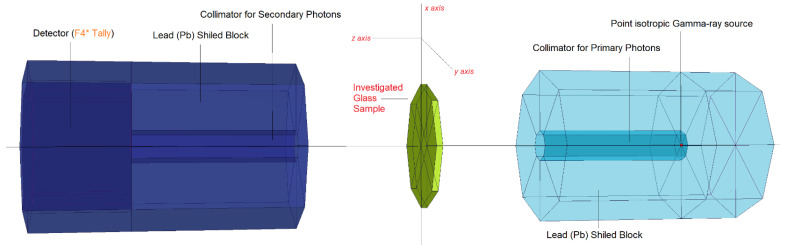
3-D model of the modelled MCNPX simulation setup (obtained from VISED_X_22S).

Within a lead (Pb) shield block, a point source was positioned (see Figure 1). Following that, the glass specimens’ elemental compositions (in weight percent) and densities (in grams per cubic centimeter) were calculated. Alternatively, as illustrated in Figure 1, the simulated point isotropic source can be considered as an extension of the overall gamma-ray transmission arrangement. The elemental mass fractions of the S1, S2, S3, S4, and S5 glasses under examination are listed in Table 1.

Notably, the MCNPX INPUT file [36,37,38] used to specify the elemental composition of glass specimens contained an *M_n_* variable. We were able to determine the importance of photon and electron interactions (IMP: p, e) in the cell using the results of the initial cell description technique. To tally the attenuated gamma rays produced, S1, S2, S3, S4, and S5 glasses were linked to the opposite side of the detection area (F4 Tally-Mesh). This type of tally mesh is advantageous for determining the average photon flux. After each run was repeated, a total of 10^8^ particles with varying photon energies were collected for each glass sample. When all simulations were run, the MCNPX model had an uncertainty rate of less than 1%. To assess the consistency of the obtained mass attenuation coefficients from the MCNPX code, we used the well-known Phy-X/PSD [39] online calculation platform. Our findings showed that both results were in good harmony in terms of their quantitative values. Accordingly, the mass attenuation coefficients of the investigated glasses were used for the determination of other critical gamma-ray shielding parameters.

## 3. Results and Discussion

**Figure 2:**
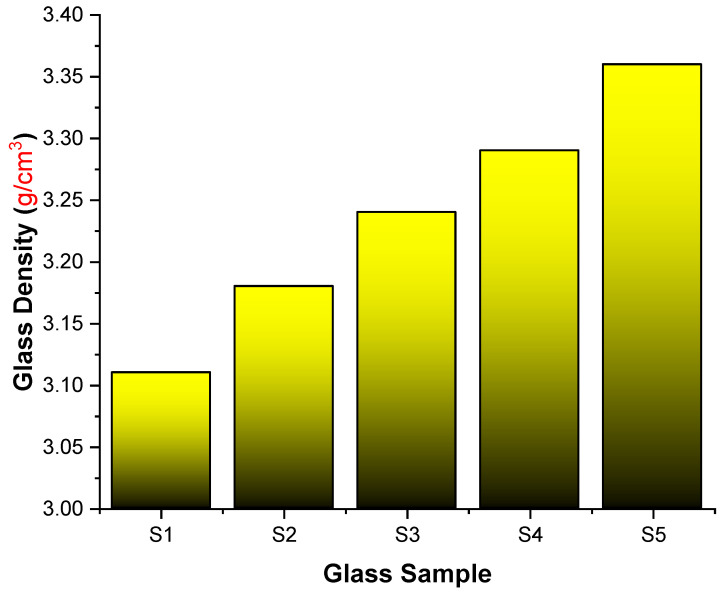
Variation of the glass density (g/cm^3^).

The gamma-ray attenuation characteristics of five glass samples strengthened with lanthanum oxide were explored in this research. Table 1 summarizes the molar and elemental mass fractions and densities of the specimens examined. We used two distinct approaches to determine the linear attenuation coefficients (μ) of the glass samples: Monte Carlo simulations and theoretical calculations. The densities of the S1, S2, S3, S4, and S5 glass specimens are shown in Figure 2. As shown in the graph, the glass density increased from 3.11 g/cm^3^ to 3.36 g/cm^3^. The S5 sample, with the highest concentration of lanthanum oxide in its structure, had the highest glass density.

**Figure 3:**
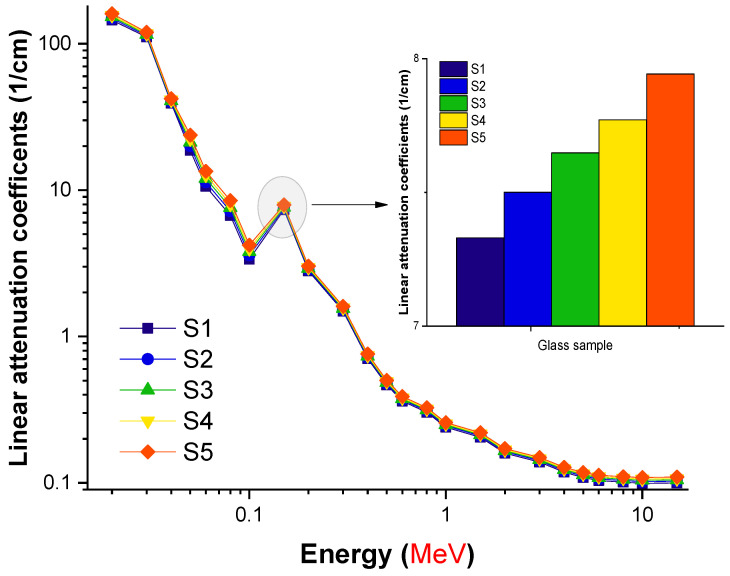
Variation in the linear attenuation coefficients (1/cm) of the investigated glasses as a function of incident photon energy (MeV).

Given that the linear attenuation coefficient is a density-dependent characteristic, it is assumed that a relation exists here between density and the linear attenuation coefficient values, and, hence, the amount of lanthanum oxide. The shifting linear attenuation coefficients (cm^−1^) as a function of the incident photon energy are depicted in Figure 3. In the graph, it can be observed that as the photon energy increased, the linear attenuation coefficients quickly decreased, reaching as low as 0.06 MeV in certain cases. Most of the photon–matter interactions occur in the low-energy area, where the photoelectric effect dominates, with cross-sectional changes proportional to *Z*^(4–5)^. The lowest-energy region exhibits the highest linear attenuation coefficients. Furthermore, the incoming photon energy, shown by the symbol E^3.5^, is well defined and inversely proportionate. However, the pair creation process dominates, and the cross section for this process is connected to *Z*^2^ at energies greater than 1.022 MeV; as a result, it was discovered that the μ values increased somewhat in this area. Taking into consideration the variations in chemical composition of the glass specimens, Compton scattering becomes increasingly significant for medium-level energies beyond the energy level of 0.06 MeV. Since there is a linear link between the cross section of Compton scattering and atomic number *Z*, the μ values of the glasses declined slowly and were stable below 1 MeV. Nevertheless, as the glass density changed gradually, no significant variations in the linear attenuation coefficients were seen. We observed an interesting effect of lanthanum oxide on the photon resistance of glass samples at various energies. Our results reveal that the S5 sample, which contained the highest concentration of lanthanum oxide, had the maximum linear attenuation coefficients for all entering photon energies. This is explained by the S5 sample’s glass density, containing the largest amount of lanthanum oxide in the glass structure. Meanwhile, another critical statistic for gamma-ray shielding, namely, the mass attenuation coefficient (μ_m_), was calculated.

**Figure 4:**
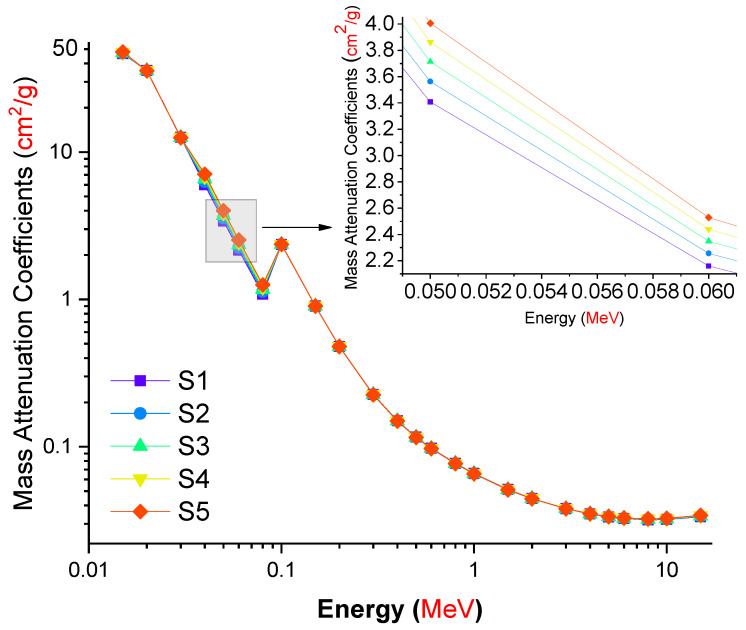
Variation in the mass attenuation coefficients (cm^2^/g) of the investigated glasses as a function of incident photon energy (MeV).

Figure 4 depicts the shifting trend in mass attenuation coefficients as a function of incoming photon energy. When the energy was increased from 0.015 MeV to 15 MeV, the mass attenuation coefficients dropped. This might be a result of difficulties encountered during the absorption of high-energy gamma rays with a large penetration factor. On the other hand, S5 exhibits the greatest m values over the whole gamma-ray energy range studied. This might be explained by the fact that S5′s glass structure had the largest amount of La (see Table 1). Apart from increasing the glass density of S5, our results indicated that around 5.12 weight percent La reinforcement enhanced the elemental characteristics of S5 in terms of density-independent gamma-ray attenuation, namely, the mass attenuation coefficient (μ_m_).

**Figure 5:**
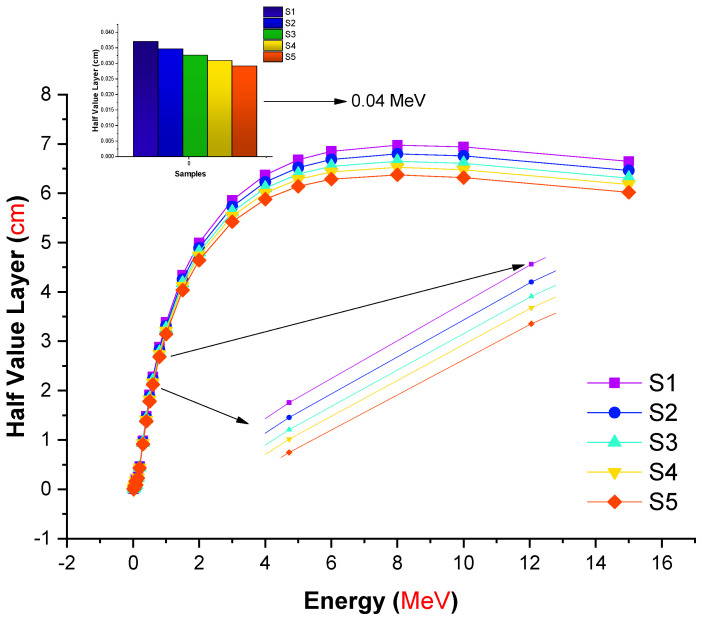
Variation in the half value layer (cm) values of the investigated glasses as a function of incident photon energy (MeV).

The half value layer term (also known as the HVL) is significant in radiation shielding research since it allows for the quantification of the material thickness required to halve the initial gamma-ray intensity. This is because radiation studies necessitate that shielding requirements be determined in advance based on the type and energy of the radiation used. As a result, the amount of the half value layer required for each type of prospective shielding material should be determined on the basis of a more complete understanding of gamma-ray attenuation capabilities during the incident gamma ray’s contact with the attenuator specimen. The fluctuation trend of the half value layer (cm) values of the examined glasses as a function of incident photon energy is depicted in Figure 5. As expected, the required half value layer grows with increasing gamma-ray energy. This is a frequently seen effect of increased gamma-ray energy and, hence, penetrating dominance of accompanying gamma-ray photons. In another sense, larger shields may be capable of deflecting powerfully penetrating gamma rays. Our findings indicate that the S5 sample meets the absolute minimum requirements for glass thickness. This is yet another strong indication of the S5 sample’s superior gamma-ray shielding characteristics due to the highest contribution of lanthanum oxide. Another significant parameter in the radiation shielding field is the tenth value layer (TVL), which is the material thickness needed to attenuate 10% of the initial incident gamma-ray intensity.

As shown in Table 2, the half value layer (T_1/2_) values of the investigated S5 samples were compared with those of Glass1 (Cr_2_O_3_-doped BS glass) [40], Glass2 (obsidian glass doped with CeO_2_) [41], Glass3 (BaO-TiO_2_-SiO_2_-B_2_O_3_ glass) [42], Glass4 (Li_2_O-K_2_O-B_2_O_3_-PbO glass) [43], Glass5 (Li_2_O-K_2_O-B_2_O_3_-HMO (HMO = SrO/TeO_2_/PbO/Bi_2_O_3_)) [44], Glass6 (xBaO-(0.30-x) MgO-0.10Na_2_O-0.10Al_2_O_3_-0.50B_2_O_3_ glass) [45], and standard shielding materials (ordinary concrete: OC [46], and hematite-serpentine concrete: HSC [47]); the T_1/2_ values of the S5 samples were lower than those of all other samples, even OC and HSC.

**Figure 6:**
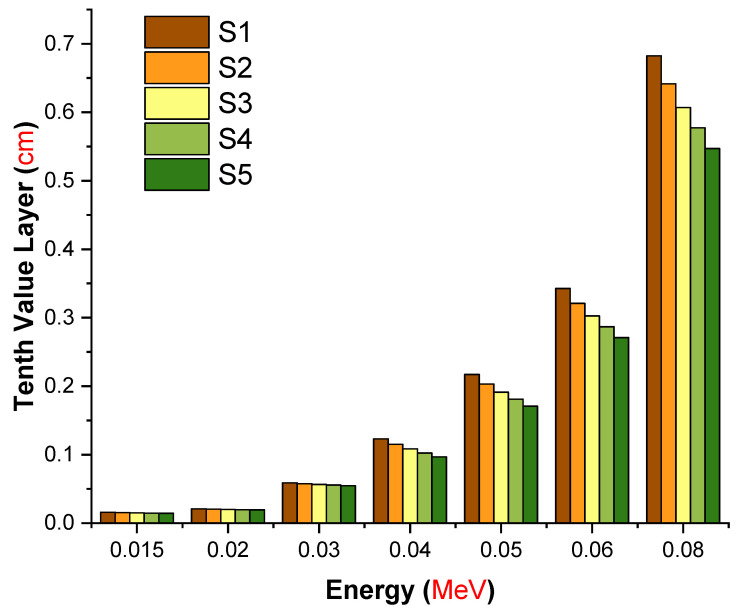
Variation in the tenth value layer (cm) values of the investigated glasses as a function of incident photon energy (MeV).

Figure 6 depicts the variation of the TVL in centimeters of the investigated glasses at selected energy levels. From Figure 6, it is clear that the change in TVL for all studied glasses (S1–S5) with low energy was small and their values tended to be close to zero in accordance with the photoelectric effect (PE) cross section dominance. With increasing energy, the values of TVL were enhanced due to the dominance of both processes of Compton scattering (CS) and pair production (PP) interactions. The S1 glass sample with ρ = 3.11 g/cm^3^ had the maximum TVL values, while the S5 glass sample with ρ = 3.36 g/cm^3^ had the minimum values at all selected energies. Therefore, the TVL values of the investigated glasses exhibited a reverse trend to the µ_m_. Thus, (TVL)_S1_ > (TVL)_S2_ > (TVL)_S3_ > (TVL)_S4_ > (TVL)_S5_. This result confirms that the S5 sample can be considered superior for gamma-ray shielding among the investigated samples. Gamma rays, upon reaching the atomic structure, are projected to engage progressively with the electrons of attenuator materials. This is a natural way for incident energy to be dispersed. However, the mean distance traveled by the gamma rays between two sequential interactions must be known. Fortunately, the mean free path (MFP) is a convenient parameter that concisely defines the mean distance between two sequential encounters. In other words, a smaller mean free path indicates a better shielding substance.

**Figure 7:**
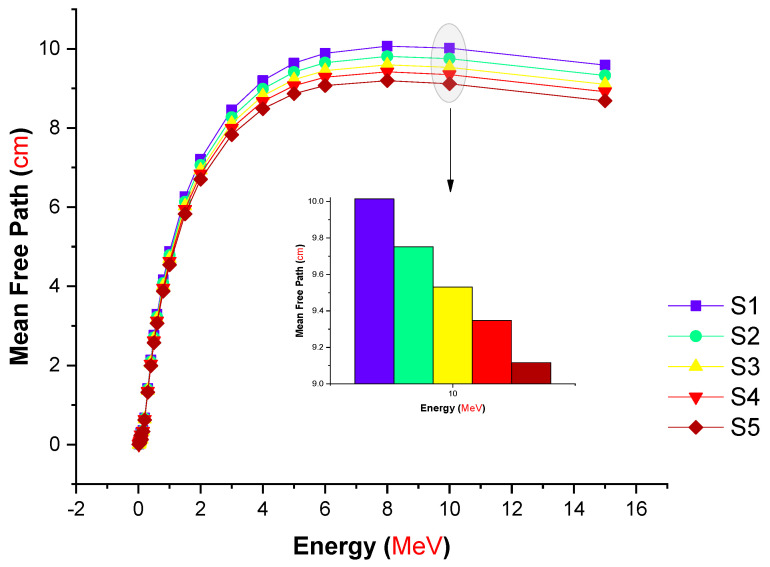
Variation in the mean free path (cm) values of the investigated glasses as a function of incident photon energy (MeV).

The difference in MFP (cm) values for the tested glasses as a function of incident photon energy is depicted in Figure 7. The MFP is a significant statistic in the field of radiation research, especially in studies of radiation shielding. This is because the findings provide unique information in terms of a more accurate estimate of the mean distance for an adjacent incident gamma ray’s interaction with the material environment. As a consequence, one may argue that decreasing the value results in a more attenuating environment for energetic gamma rays. We determined the MFP values of all the glass samples examined in this research.

**Figure 8:**
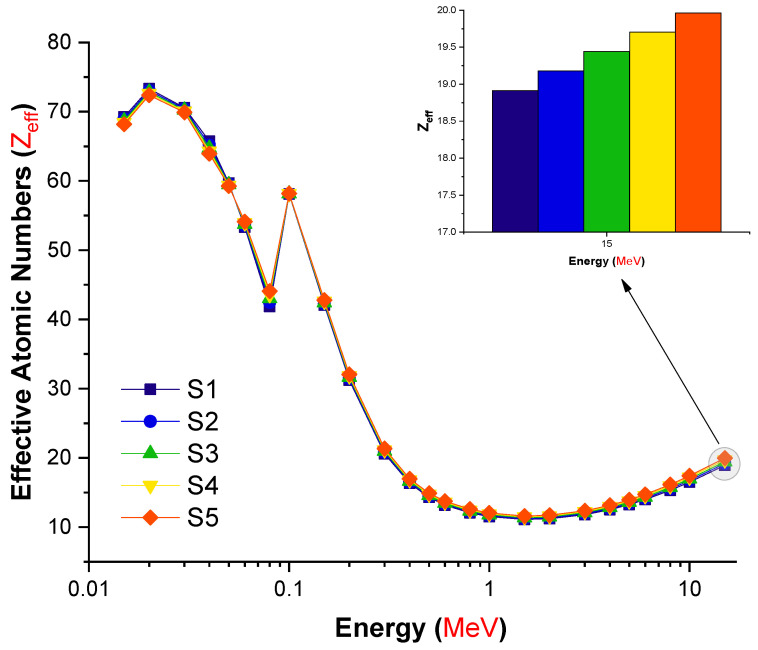
Variation in the effective atomic number (Z_eff_) values of the investigated glasses as a function of incident photon energy (MeV).

In addition to the superior gamma-ray attenuation parameters obtained for the S5 sample, a similar trend in the MFP values was observed. For the S5 sample, minimal MFP values were found at all photon energies investigated. The fluctuation in the effective atomic number (Z_eff_) values of the examined glasses as a function of incident photon energy is depicted in Figure 8.

**Figure 9:**
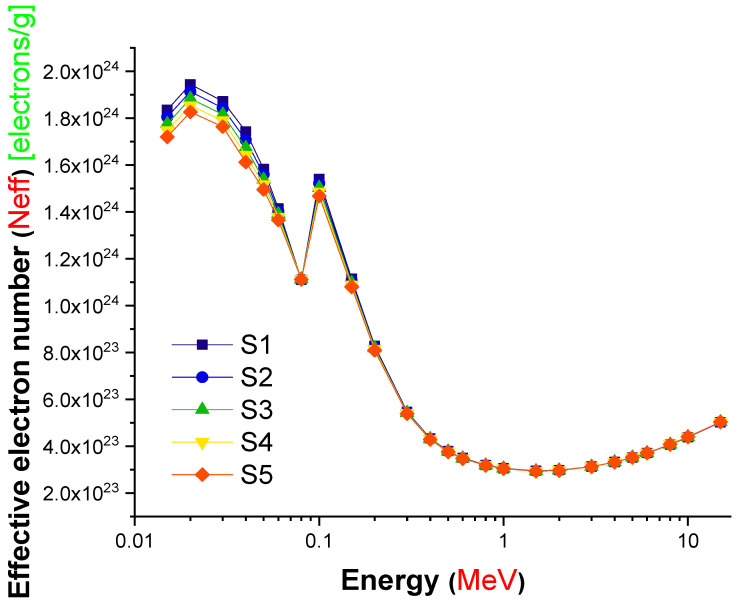
Variation in the effective electron number (N_eff_) values of the investigated glasses as a function of incident photon energy (MeV).

The effective atomic number is a critical quantity that offers precise information about the effective atomic number for attenuation at specified energy values. S5 was observed to have the maximum effective atomic number values at all energies examined, as illustrated in Figure 8. This can be explained by the increased amount of lanthanum oxide reinforcement, which increased the glasses’ overall atomic number from S1 to S5. As a result, the S5 sample’s total atomic number changed significantly due to differences in the glass structure between the reduced (B_2_O_3_) and increased (L_2_O_3_) replacements. Additionally, the average atomic number of the two substitute materials can be determined. The variation in the net atomic numbers of the glasses tested is the primary explanation for the differences in their effective numbers. Our results reveal that the S5 sample had the highest Z_eff_ values over the whole range of gamma-ray energy. Additionally, the effective electron density (N_eff_), which presents the number of electrons per unit mass, was evaluated for the S1–S5 glasses. The change in the effective electron number (N_eff_) values of the S1–S5 glasses is shown in Figure 9 as a function of incident photon energy (MeV).

**Figure 10:**
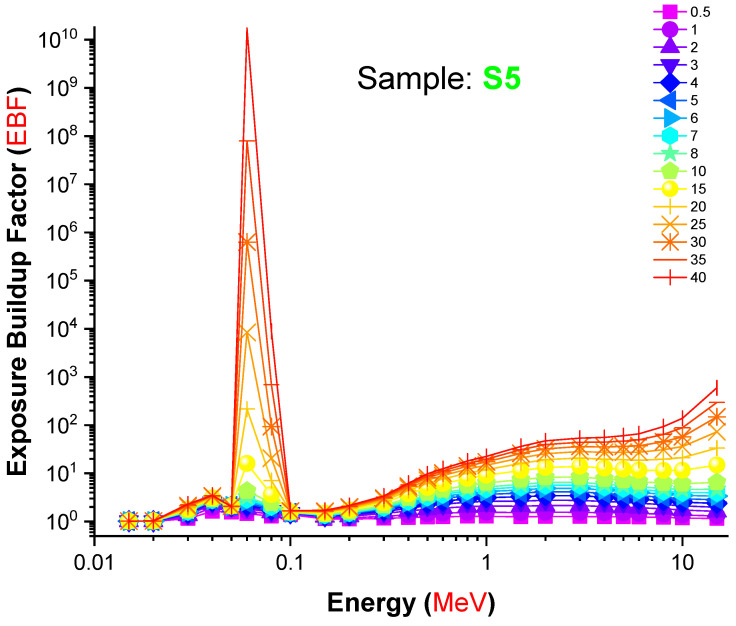
Variation in the exposure build-up factor (EBF) values of the S5 glass sample as a function of incident photon energy (MeV).

**Figure 11:**
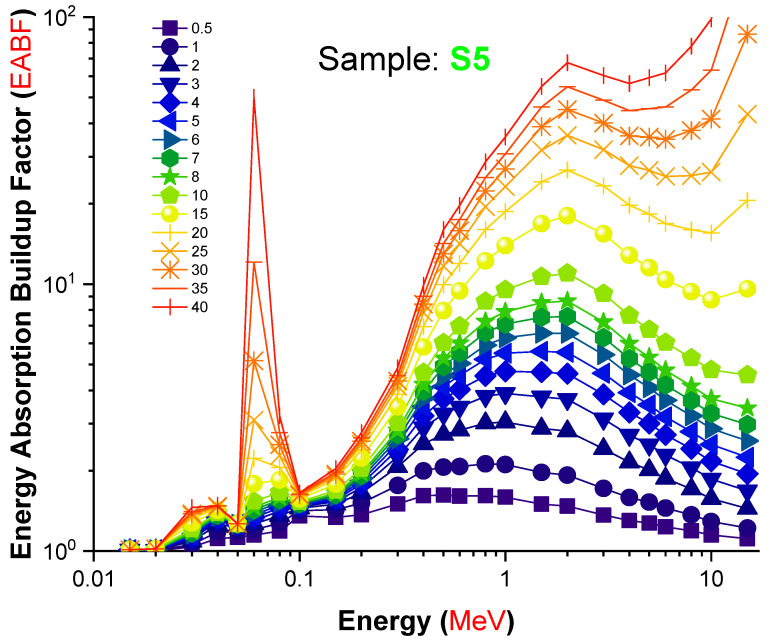
Variation in the energy absorption build-up factor (EABF) values of the S5 glass sample as a function of incident photon energy (MeV).

From Figure 9, in the low region of photon energy, N_eff_ changed in a non-monotonic trend with photon energy until reaching a sudden jump near the absorption edge of Pb (0.0880 MeV). This behavior can be attributed to the PE process. In the photon energy zone from 0.1 to 1 MeV, a quick decrease in the N_eff_ values was observed in all investigated samples; this trend is related to the CS process, which dominates in this region. In the energy zone greater than 2 MeV, an increase in the N_eff_ values was observed and attributed to the PP process, which dominates in this region. Our data indicate definitively that the S5 sample, which contained the highest concentration of lanthanum oxide, had a significant advantage in terms of gamma-ray attenuation. A total of 2 mole percent replacement of B_2_O_3_ for L_2_O_3_ resulted in a 0.23 g/cm^3^ density shift. Furthermore, this modification altered the gamma-ray shielding properties of the glass samples evaluated. The term “Build-up factor” is required for an effective evaluation of gamma attenuation and may affect the measurement’s quality. Gamma ray measurement is required for nuclear technology since it is used in industry, medicine, agriculture, education, research, and military applications. Additionally, it is necessary for the building of radiation protective structures that protect human health. When gamma radiation travels via shielding material, two types of radiation are produced: un-collided photons and colliding photons. As a result, the accumulation factor is an essential statistic for gamma ray measurement. It is defined as the ratio of the total number of particles at a given point to the total number of particles that have not collided at that location. Figure 10 and Figure 11 illustrate the variation in the EBF and EABF values for S5 glass samples. Gamma rays are absorbed mostly in the low- and high-energy bands, which account for the majority of absorption. On the other hand, at intermediate energies, Compton scattering is the dominant mechanism of photon–matter interaction. As a result, in the low-energy zone, the Compton region has the greatest EBF values. Not only was the S5 sample deficient in terms of overall EBF, it was also the only sample with regional variance in EABF. S5 had the lowest EBF and EABF values across the whole photon energy and penetration depth range, according to our findings.

**Figure 12:**
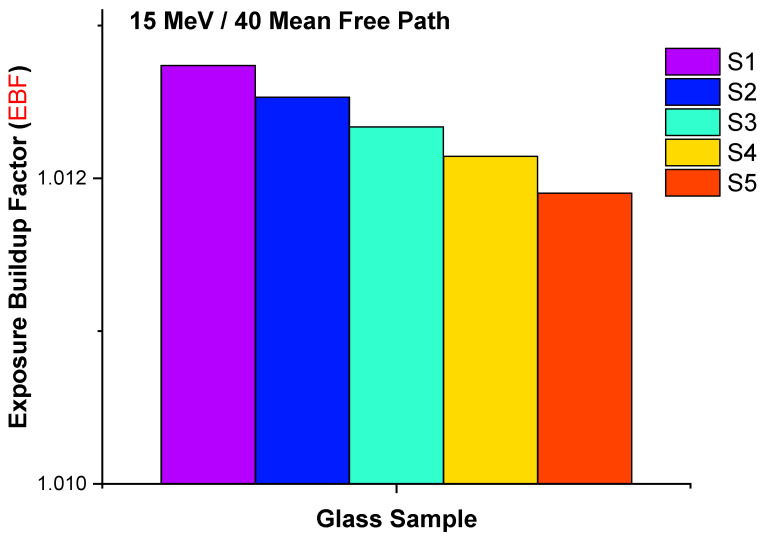
Differences in the exposure build-up factor values of the S1, S2, S3, S4, and S5 samples for 40 mean free path at 15 MeV photon energy (MeV).

A closer look at the variation in the EBF values of all glass samples at 15 MeV for 40 MFP is also presented in Figure 12. It can be seen that the EBF values decreased linearly from sample S1 to S5 at 15 MeV for 40 MFP. One may conclude that the number of colliding photons grew as the glass density and lanthanum content rose. This is also explained by the direct influence of increasing lanthanum oxide reducing EBF values and thereby increasing the gamma-ray attenuation capabilities of the examined glasses. However, as seen from Figure 12, the mean differences were not so high. This can be explained by the similar elemental compositions and densities of the glass samples. However, our results indicated that increasing La contribution had a monotonic effect on all gamma-ray shielding parameters.

Finally, it is important to discuss the verification of the obtained findings, which were utilized to calculate the gamma-ray shielding parameters for the S1, S2, S3, S4, and S5 glasses. To begin, the mass attenuation coefficients of the examined glasses were computed using the MCNPX general-purpose code, as detailed in earlier sections. To ensure the integrity of our findings, we compared the mass attenuation coefficients obtained via MCNPX to those calculated using standard NIST data, Phy-X/PSD. Table 3 compares the mass attenuation coefficients determined for various energies. As can be seen, there is a high degree of numerical consistency for each particular energy value. However, we discovered some minor variations between the values from MCNPX and Phy-X/PSD. This difference may be related to the tools’ conceptual differences, with MCNPX being an input-based simulation code and Phy-X/PSD being an online computation platform. In other words, a simulation environment and associated tools should be created in MCNPX code, together with their associated characteristics (see Figure 1). The dimensions chosen, the variance reduction strategies employed, and the general performance of the computer may all have an effect on the overall simulation. On the other hand, the libraries and physics lists that are utilized may have an effect on the numerical results. As shown in Table 3, however, mass attenuation coefficients were reported with a high degree of correlation. On the other hand, multiple prior investigations have shown the compatibility of the MCNPX simulation code with experimental results [48,49,50,51,52]. As a result, the MCNPX simulation data may be regarded to be verified with standard databases and to have a satisfactory degree of consistency.

## 4. Conclusions

In this study, we aimed to evaluate different types of gamma-ray shielding parameters using advanced simulation methods. The hypothesis of a recent simulation study was to observe an enhancement in gamma-ray shielding properties with increasing lanthanum oxide reinforcement in the glass structure. Accordingly, the influence of lanthanum oxide on the gamma-ray shielding properties of cobalt-doped heavy metal borate glasses with chemical formula 0.3CoO-(80-x)B_2_O_3_-19.7PbO-xLa_2_O_3_: x = 0, 0.5, 1, 1.5, and 2 mole% was examined using MCNPX general purpose Monte Carlo code and the Phy-X/PSD online platform. Several significant radiation shielding parameters were evaluated. The glass density was increased from 3.11 to 3.36 g/cm^3^ with increasing La^3+^ ion content from 0 to 2 mole%. The mass attenuation coefficients for all glasses were evaluated via the MCNPX code and Phy-X/PSD online calculation platform. Our findings showed that both results were in good harmony in terms of their quantitative values. The S5 glass sample, which contained the highest concentration of La^3+^ ions (2 mole%), had the maximum linear (μ) and mass (μ_m_) attenuation coefficients for all entering photon energies, while the S1 glass sample, free of La^3+^ ions, possessed the minimum values of μ and μ_m_. The half value layer (T_1/2_), tenth value layer (TVL), and mean free path (MFP) of all investigated glasses showed a similar trend of (T_1/_2, TVL, MFP)_S1_ > (T_1/2_, TVL, MFP)_S2_ > (T_1/2_,TVL, MFP)_S3_ > (T_1/2_,TVL, MFP)_S4_ > (T_1/2_, TVL, MFP)_S5_. Our results revealed that the S5 sample had the highest effective atomic number (Z_eff_) values over the whole range of gamma-ray energy. S5 had the lowest EBF and EABF values across the whole photon energy and penetration depth range. Our simulation findings give a strong indication of the S5 sample’s superior gamma-ray shielding characteristics due to its highest contribution of lanthanum oxide. On the other hand, it can be concluded that advanced simulations for radiation transport studies such as Monte Carlo simulations can be utilized for an initial assessment of candidate glass shields to determine their gamma-ray attenuation properties before manufacture.

## Figures and Tables

**Table 1:** Elemental mass fractions (wt.%) and densities of the glasses [10].

Element	S1	S2	S3	S4	S5	Density (g/cm^3^)
Co	0.0018	0.0017	0.0017	0.0017	0.0017	3.11
O	0.4164	0.4112	0.4060	0.4010	0.3961	3.18
B	0.1732	0.1699	0.1667	0.1636	0.1606	3.24
Pb	0.4086	0.4035	0.3984	0.3935	0.3887	3.29
La		0.0137	0.0271	0.0402	0.0529	3.36

**Table 2:** Comparison of the half value layer (cm) values of the investigated S5 glass sample, different glass samples, and standard shielding materials as a function of incident photon energy (MeV).

Energy MeV	Cr_2_O_3_ Doped BS Glass	Obsidian Glass Doped with CeO2	BaO-TiO_2_-SiO_2_-B_2_O_3_ Glass	Li_2_O-K_2_O-B_2_O_3_-PbO Glass	Li_2_O-K_2_O-B_2_O_3_-HMO (HMO = SrO/TeO_2_/PbO/Bi_2_O_3_)	xBaO-(0.30-x) MgO-0.10Na_2_O-0.10Al_2_O_3_-0.50B_2_O_3_ Glass	OC	HSC	ILC	S5
0.015	0.06999	0.01193	0.02506	0.01789	0.04208	0.01431	0.04339	0.01311	0.00819	0.00431
0.02	0.16263	0.02700	0.05557	0.02694	0.03728	0.03155	0.10046	0.02950	0.01844	0.00578
0.03	0.48514	0.08548	0.16504	0.07857	0.11118	0.09529	0.30814	0.09301	0.05865	0.01645
0.04	0.90467	0.18801	0.09281	0.16464	0.23530	0.03963	0.60803	0.20492	0.13169	0.02912
0.05	1.29146	0.33098	0.16032	0.28368	0.40383	0.07014	0.92174	0.36290	0.23959	0.05149
0.06	1.59083	0.45375	0.24766	0.42856	0.60044	0.11223	1.19271	0.55220	0.37682	0.08159
0.08	1.97435	0.78133	0.46435	0.75322	1.00373	0.23103	1.57142	0.94984	0.69461	0.16469
0.1	2.20245	1.06830	0.70521	0.42251	1.34681	0.39192	1.80291	1.29198	1.00007	0.08727
0.15	2.55314	1.54137	1.23868	0.90447	1.90600	0.88913	2.14202	1.84225	1.54005	0.22900
0.2	2.81264	1.81964	1.61220	1.37635	2.23682	1.36937	2.37485	2.16102	1.85716	0.43151
0.3	3.24638	2.18700	2.08431	2.09286	2.68077	2.07147	2.74889	2.58396	2.25913	0.91485
0.4	3.62308	2.46855	2.41137	2.58742	3.02579	2.53654	3.07150	2.91330	2.55906	1.38074
0.5	3.96650	2.71454	2.67917	2.96740	3.32676	2.89115	3.36401	3.20123	2.81693	1.78212
0.6	4.28767	2.94004	2.91751	3.28797	3.60324	3.18990	3.63650	3.46617	3.05218	2.12452
0.8	4.87972	3.35196	3.34302	3.83417	4.10858	3.70145	4.13916	3.95012	3.48116	2.68617
1	5.42603	3.73004	3.72850	4.31099	4.57193	4.15112	4.60294	4.39535	3.87447	3.14895
1.5	6.65908	4.57423	4.58515	5.33685	5.61216	5.11781	5.64834	5.38995	4.74804	4.03937
2	7.72495	5.28429	5.31191	6.17919	6.50486	5.89570	6.54876	6.22634	5.47198	4.64420
3	9.51059	6.41277	6.50536	7.52587	7.98139	7.08310	8.04572	7.56504	6.59537	5.42648
4	10.93855	7.25804	7.44252	8.55353	9.14832	7.93067	9.23181	8.57325	7.40447	5.88173
5	12.10378	7.89853	8.19022	9.35246	10.08779	8.54238	10.19199	9.33846	7.99386	6.14438
6	13.05219	8.38415	8.78821	9.97226	10.83892	8.98621	10.96492	9.92337	8.42200	6.28726
8	14.47857	9.03085	9.66371	10.83906	11.95002	9.53598	12.10822	10.71325	8.95527	6.37191
10	15.45685	9.40454	10.23140	11.36501	12.68374	9.80251	12.87456	11.17529	9.22487	6.31923
15	16.81245	9.76914	10.95281	11.93931	13.65135	9.93753	13.89992	11.63975	9.40269	6.01955

**Table 3:** A numerical comparison of the mass attenuation coefficients of S1, S2, S3, S4, and S5 glasses obtained from Phy-X/PSD and MCNPX.

	S1		S2		S3		S4		S5	
Energy (MeV)	Phy-X/PSD	MCNPX	Phy-X/PSD	MCNPX	Phy-X/PSD	MCNPX	Phy-X/PSD	MCNPX	Phy-X/PSD	MCNPX
0.015	46.550	47.795	46.884	47.985	47.210	47.324	47.528	18.128	47.838	48.120
0.02	35.757	36.124	35.732	36.251	35.708	35.802	35.684	36.123	35.661	35.684
0.03	12.598	13.058	12.582	13.124	12.567	12.615	12.553	12.625	12.539	12.541
0.04	6.014	6.125	6.291	6.325	6.562	6.594	6.826	6.914	7.084	7.106
0.05	3.407	3.512	3.563	3.592	3.714	3.728	3.862	3.895	4.006	4.108
0.06	2.160	2.174	2.256	2.271	2.349	2.351	2.440	2.459	2.528	2.529
0.08	1.085	1.108	1.129	1.156	1.171	1.180	1.212	1.135	1.253	1.256
0.1	2.357	2.412	2.359	2.415	2.360	2.417	2.362	2.401	2.364	2.403
0.15	0.902	0.921	0.902	0.923	0.901	0.925	0.901	0.927	0.901	0.931
0.2	0.480	0.497	0.479	0.499	0.479	0.501	0.478	0.503	0.478	0.509
0.3	0.227	0.233	0.226	0.235	0.226	0.237	0.226	0.238	0.225	0.241
0.4	0.150	0.159	0.150	0.161	0.150	0.163	0.150	0.165	0.149	0.168
0.5	0.116	0.124	0.116	0.126	0.116	0.127	0.116	0.129	0.116	0.131
0.6	0.098	0.109	0.097	0.110	0.097	0.112	0.097	0.115	0.097	0.117
0.8	0.077	0.081	0.077	0.082	0.077	0.083	0.077	0.085	0.077	0.089
1	0.066	0.070	0.066	0.072	0.066	0.073	0.066	0.074	0.066	0.076
1.5	0.051	0.054	0.051	0.056	0.051	0.058	0.051	0.060	0.051	0.062
2	0.045	0.047	0.045	0.047	0.044	0.049	0.044	0.051	0.044	0.053
3	0.038	0.040	0.038	0.041	0.038	0.042	0.038	0.043	0.038	0.045
4	0.035	0.038	0.035	0.039	0.035	0.041	0.035	0.042	0.035	0.043
5	0.033	0.035	0.033	0.035	0.033	0.035	0.034	0.035	0.034	0.036
6	0.033	0.034	0.033	0.034	0.033	0.035	0.033	0.036	0.033	0.037
8	0.032	0.034	0.032	0.035	0.032	0.036	0.032	0.037	0.032	0.037
10	0.032	0.031	0.032	0.032	0.032	0.033	0.033	0.033	0.033	0.035
15	0.034	0.036	0.034	0.036	0.034	0.037	0.034	0.038	0.034	0.039

## Data Availability

The data presented in this study are available on request from the corresponding author.
